# The Value of Dignity in Prison: A Qualitative Study with Life Convicts

**DOI:** 10.3390/bs10060095

**Published:** 2020-05-28

**Authors:** Ines Testoni, Francesca Marrella, Gianmarco Biancalani, Paolo Cottone, Francesca Alemanno, David Mamo, Luigi Grassi

**Affiliations:** 1 Department of Philosophy, Sociology, Education and Applied Psychology (FISPPA), University of Padova, 35122 Padova, Italy; francescamarrella@outlook.it (F.M.); gianmarco.biancalani@unipd.it (G.B.); paolo.cottone@unipd.it (P.C.); 2 Emili Sagol Creative Arts Therapies Research Center, University of Haifa, Haifa 3498838, Israel; 3 European and Mediterranean Cultures (DiCEM) Department, University of Basilicata, 85100 Potenza, Italy; francesca.alemanno@unibas.it; 4 General Adult & Geriatric Psychiatry, Faculty of Medicine, University of Malta, 2080 Msida MSD, Malta; david.mamo1@gmail.com; 5 Institute of Psychiatry, Department of Biomedical and Specialty Surgical Sciences—Università di Ferrara, 44121 Ferrara, Italy; luigi.grassi@unife.it

**Keywords:** dignity, prisoners, men, life sentence, personal values

## Abstract

Background: This research is based on the perspective of dignity according to Chochinov; thus, the life imprisonment of detainees is assimilated to a severe disease. Methods: Ten male prisoners were interviewed trough Chochinov’s Dignity Therapy, and the results were analysed using thematic analysis. Results: Two areas of thematic prevalence emerged, namely, value of freedom, self-consciousness and education and their failure in jail, and life sentence as annihilation of life meaning and of the values of generativity and family. Conclusions: Life imprisonment has been described in its negativity by several respondents as a punishment worse than the death penalty. It has been compared to death itself, to a terminal illness, to torture and to a pain that grows over the years, with the awareness that despite the passing of time, you will not have the opportunity to return to your loved one and to a free life. In fact, prisoners live out their condition within a space in which any value that gives meaning to life risks being destroyed.

## 1. Introduction 

Life imprisonment is the punishment of being put in prison without any release arrangements or until death. The Grand Chamber of the European Court of Human Rights (ECHR) affirmed that the very essence of the ECHR is to recognise and protect human dignity [1]. In relation to prisoners sentenced for life, this involves providing a realistic possibility of release [2,3]. In keeping with this, the European Prison Rules underlined that prison regimes should be designed to enable all sentenced prisoners to lead a responsible and crime-free life [4]. The ECHR has emphasised the importance of giving every offender the opportunity to rehabilitate whilst serving his/her sentence, with the prospect of eventually functioning as a responsible member of free society [2]. According to Shannon [5], dignity is a quality or state of being worthy, honoured or esteemed, and it is ‘realized through individual freedom that is brought to bear in the course of the self’s participation in meaningful decision making and exercise of individual responsibility’ [5] (p. 17). 

The concept of dignity has been used in many human studies. In philosophy, the concept of dignity and respect of person is highlighted in the second categorical imperative theorised by Kant: ‘Act in such a way that you treat humanity, whether in your own person or in the person of any other, never merely as a means to an end, but always at the same time as an end’. In fact, with this imperative, the absolute dignity of the human person, which derives from reason, is affirmed. Sensitive impulses and natural inclinations must be subordinated to this so that rationality becomes the end of conduct and not the means to achieve utilitarian purposes, to which reason itself would be subservient. In bioethics, dignity is understood as (1) the grounding value of human life and (2) is applicable to medical practice [6]. As such, the American Medical Association has cited dignity as the first of its nine principles [7]. In forensic psychiatry, the implications of dignity have been discussed in ongoing debates: some experts claim that it is not just a principle that leads individuals to respect others’ autonomy, but a principle that needs to be used in a more general – but precise – way [8]. The concept of dignity, as something a judge provides to a convicted person, is also used in procedural justice to promote compliance with the law during and after court supervision [9]. Thanks to Chochinov’s ‘Dignity Therapy’ (DT) [10], the word ‘dignity’ has been used more frequently in ‘end of life’ contexts, insofar as a life-threatening illness often leads to a loss of independence, identity and dignity [11,12]. 

Indeed, the human dignity issues we see in life-sentenced prisoners are fostered by their fear of deteriorating in prison. Moreover, men become increasingly introverted as their sentence progresses, and they express less interest in social activities and ‘outgoing’ behaviour [13,14,15]. With regard to Italian law, the life sentence [16] (art. 17, n. 2) is never-ending, and it has to be served in one of the designated areas, supplemented with work duties and nocturnal isolation [16] (art. 29, 32, 36, 72). A person who is serving a life sentence can strive for three ‘prizes’: additional authorisations, parole and conditional release. To access these prizes, they must maintain appropriate conduct, be excluded from the category of ‘social dangerousness’ and demonstrate willingness to develop cultural, affective or working interests. It is also essential to have served at least 10 years of the sentence [17]. 

Specifically, there are different types of life sentence, among which is the so-called ‘ergastolo ostativo’ or irreducible life sentence, which applies specifically to criminal violations. The convicts who are part of this group have to collaborate with officials to gain access to the above-mentioned prizes; otherwise, the special detention regime 41, comma 2, expounded from the law nr. 354 of year 1975 of the penitentiary set of rules may take effect [18]. This treatment is actualised only to protect the safety of civilians. It applies only to prisoners who exhibit high criminal risk and those who could, theoretically, pursue their goals through their criminal contacts in prison. This type of imprisonment regime is tailor-made to prevent mafia-related crimes as mafia members can still maintain connections with their criminal group outside prison. Faced with these realities once can appreciate how life imprisonment has the potential to negatively impact meaning and dignity of the prisoner. Chochninov’s dignity-conserving care model [19] designed for use in a palliative care setting may shed light on the subjective experience of dignity of a prisoner with a life sentence. A related mediator and motivator of meaning in human experience is the concept of ‘value’. Like Chochninov’s ‘dignity conserving care model’, Schwartz’s matrix of values [20,21]. Both these perspectives share the idea that the meaning of life can be understood by considering the relationships between values and past experiences. Schwartz’s Theory of Basic Human Values (TBHV), which identifies 10 human value orientations [20,21]. These orientations are arranged along a motivational continuum, forming a circular structure wherein adjacent values are compatible with each other, whilst those opposite in the circle are contrary to each other [20]. Chochinov [19] focused his research on the idea of ‘dignity’, which can be considered as the main value. In this view, dignity is composed of respect and recognition of the constellation of all unalienable personal values. We wanted to consider the possibility that life prisoners also carry a dignity, which must be respected through the recognition of the values that give their life meaning. Indeed, as indicated by the TBHV, values constitute the basis of attitudes and behaviours in all cultures. However, the condition of imprisonment can deeply interfere with these values. 

The experience of total pain, which characterises terminal illnesses, does not consist only in physical suffering, but also in existential suffering due to the loss of future prospects and life meaning as life comes to an end [22,23,24]. The psychological dimension of this expression of human suffering is linked to the loss of one’s sense of generativity or “legacy”, which is closely related to one’s perception of dignity. Indeed, the drive for legacy has been acknowledged especially in research exploring dignity at the end of life by Chochinov [22,23]. In Chochinov’s opinion, the transmission of the “ultimate legacy” provides a way for continuity as it emerges from the past, develops in the present of people who do not perceive their future, and gives back them a future perspective. The ultimate legacy goal of transmitting that which is most important to the individual is grounded in the values recognized by the individual. The primary task of this perspective is tooted in determining one’s most cherished values and actively conveying these values to friends, family members and/or communities. The legacy transmission perspective is comprised of sharing and giving values and meaning of life resulting from life experiences. A life sentence has the potential to rob the convict of their dignity through a perception of loss of future. In Chochinov’s opinion, three factors can influence the perception of dignity in the context of terminal illness which can resemble the TBHV: Illness-Related Concerns (e.g., physical or psychological distress, etc.), the Dignity Conserving Repertoire (e.g., continuity of self, role preservation, hopefulness, resilience, etc.); and Social Dignity Inventory (e.g., privacy boundaries, social support, care tenor, burden to others and aftermath concerns) [10]. DT is a well-researched intervention that can enhance the well-being of people dealing with life-limiting medical conditions [10] (p. 78). Given that both life sentence and terminal disease share similar attributes relating to loss of dignity and perception of meaning and value, in the present study we sought to conduct a qualitative study of meaning and values un persons serving a life sentence provided with Dignity Therapy. The therapeutic treatments administered to hospitalised patients often lead to a loss of dignity, just as behavioural interventions do for life-sentenced prisoners, and indeed these two conditions are commonly characterised by the dissolution of identity and by a loss of the factors that preserve a sense of dignity [10,11,12,13,14,15,16,17,18,19,24,25,26]. People in jail cannot be rehabilitated if there is little or no opportunity to develop their dignity through, in particular, employment, study and the maintenance of positive relationships. From this perspective, the present study followed another project realised in Italy by Perito [27], who adopted Chochinov’s DT protocol in a detention centre to improve the preservation of dignity and ameliorate the existential wellbeing of prisoners. As indicated by Chochinov, DT relieves psychological and existential distress in people living with life-threatening behaviours or life-limiting diseases and in a population without serious diseases [28]. According to Perito [27], DT can represent an ethical will, a life review and a personal narrative promoting spiritual and psychological well-being and improving the life experience of a person subject to restraint. In this research, we share Perito’s perspective, which offers prisoners the opportunity to perceive themselves through DT as people who can indicate their values to others, helping them to keep their sense of dignity alive. 

## 2. Materials and Methods

### 2.1. Aims

Using the Dignity Therapy Protocol (DTP) [10], the primary aim of this study was a qualitative examinations values and life-meaning of prisoners based on the categories of the fundamental values indicated by TBHV. The secondary aim was to consider the role of generativity of the “ultimate legacy” in relationship with values [20,21]. 

### 2.2. Participants

The study was carried out in a prison in Northern Italy with 10 male prisoners serving a life sentence. This research was held in this prison because the research team had been developing a collaboration with the structure. In fact, other studies and projects were in course and programmed for the future. For this specific activity, prisoners were selected based on their motivations, their interests in previous activities (e.g., training projects with schools, theatre or newspaper editorial staff inside the prison) and their personal developmental maturation process. After the approval of the ethics committee and the authorisation of the prison director, the prison educator proposed the project to those who were considered suitable by the psycho-socio-pedagogical team.

These participants came from different Italian regions and had different stories and reasons for their imprisonment. The research was realised in the prisoners’ natural setting in order to elicit more vivid descriptions [29] (p.3). The interviews took place in a dedicated space, closed by a door. No penitentiary staff were present inside, allowing us to freely access each participant’s memories and to protect their privacy. Each interview lasted about two hours. Participants signed an informed consent form after receiving clarification about the purpose of our research and confirmation that they could withdraw from the research at any time without consequence. At the end, a second meeting was conducted to present the results of the research. 

### 2.3. The Instruments and the Qualitative Method

The research adopted the DTP (Table 1) composed by 11 questions, including the following: ‘Tell me a little about your life history, particularly those parts that you either remember most or think are the most important; Are there any specific things that you would want your family to know about you and are there particular things you would want them to remember? What are your most important accomplishments, and what do you feel most proud of?’. The interviews were not audiotaped due to the prison’s regulations. However, all answers were recorded by the interviewer. We analysed the resulting texts using thematic analysis, which allows researchers to identify relevant issues emerging through the dialogue. Our analysis focused on the relationships between prisoners’ values and their feelings about their lives, employing critical, theory-driven interpretations based on Schwartz’s [20] and Chochinov’s works [19]. In particular, we adopted the 10 basic and transcultural values [30] that derive from one or more of the three needs of human existence: the needs of individuals as biological organisms, the requisites of coordinated social interaction and survival and welfare needs of groups [31]. The narratives were presented sequentially, although they were also sufficiently flexible to allow content-based analysis [32,33]. The process was structured following six main phases: preparatory organisation, reading and re-reading to recognise key concepts, coding data, interpreting themes, searching for alternative explanations and producing the final report [34,35]. The process proceeded in a ‘bottom-up’ manner, wherein categories only became clear as the analysis unfolded and as we explored the connections between explicit statements and implicit meanings [30,31,32,33,34,35,36,37,38,39,40]. We performed the analysis with ATLAS.ti, a computer programme that can identify the thematic networks [38,41]. The research followed APA’s Ethical Principles of Psychologists and Code of Conduct and the principles of the Declaration of Helsinki. We obtained approval from the prison’s Services Administration and the Padova University Ethics Committee for Experimentation (N. E59F91BD329B2B41F319758354BBBDB4).

## 3. Results

We used three types of reading to analyse the interviews [39]: literal reading to transcribe the interviews, interpretative reading to get an impression of the latent meaning of the interviewee’s words, and reflexive reading to finalise the interpretation of the collected data [39]. From our analysis of the transcripts, we found that certain fundamental themes arose frequently. From the analysis of the texts, two main themes emerged from the narratives: the value of freedom, self-consciousness and education and their failure in jail; and life imprisonment as the annihilation of life-meaning and of the value of generativity and the family. 

### 3.1. The Values of Freedom, Self-Consciousness and Education and Their Failure in Prison

Participants seemed to underline the value of education. Somehow, they unconsciously recalled the Socratic ideas that (1) wrongdoings and non-virtuous behaviours resulted from ignorance and (2) the most valuable knowledge is to ‘know thyself’. Paul, a married man in his forties with four sons, had been in jail in Belgium for 5 years before being imprisoned in Italy for 22 years under the special detention ‘41 bis’ regime. He said: ‘Education is important. I don’t know why, [but] here in Italy, there are few people who valorise the re-education process. It would be very significant’. Similarly, Roberto, a 62-year-old man who had spent the last 16 years in prison, placed great importance in education. In fact, he was participating in a University computer engineering course from prison: ‘I like to study, I think it’s important not to stop. I fill up all my empty time. Staying with nothing to do is stupid; you have to keep yourself occupied, mentally and practically. I should have done it before, but life wouldn’t let me. Strangely, now I can do it here, where everything of value for personal maturity seems to be denied’. Carlo, a 51-year-old man imprisoned for 23 years who had served his sentence in 11 different prisons, underlined the following ideas: 

Freedom is what gives meaning to life. Here, everything loses sense, because even when you behave correctly, you cannot go out due to your sentence. To restore value to correct behaviour, life sentence should be substituted with a temporary penalty, which allows you to obtain freedom without conditions, without measures. Education could help you to understand what is right and what is wrong. However, there are many provisions—even as a person who is serving a life sentence, it is difficult to understand them. Prisoners should be freed, and other possibilities should be given to them. 

Milosevich, who fled from former Yugoslavia in 1991, shared the following opinion: ‘It is nonsense to limit the person and his complete expression. Life sentence raises anxiety in me; there are some opportunities, but nothing can be taken for granted. When other people decide about your life, it is difficult to understand what you should know and do: everything is always a big question, and in this way, you lose the power over your life’. This pointlessness, which negates any form of value, has also been reported by two participants who lived for a couple of years under the special regime, spending 22 h a day completely alone and without any educational pursuits. Giorgio, a man in his fifties, was imprisoned for 28 years, 25 of them served in high security and 2 under the special detention regime. He said:

‘*In my opinion, life has no value here in prison. Indeed, my life was finished when I entered prison. However, I wanted to learn to paint, and the manager of the prison authorised me to have some material, but the material was bought at my own expense. […] In my opinion, learning new things is important. I try to keep growing. Although prison limits me, I try to make the most of all the opportunities that are offered to me here. Learning new things is important*’.

The limits imposed on the value of self-direction, which caused prisoners to feel that life was meaningless in prison, were also underlined by Carlo:

*The risk is to irreparably lose your freedom, credibility and personal clout. You can no longer decide anything, neither for yourself nor for others; you cannot participate in the programs organised by your family. Life sentence is comparable to being confined to a hospital even though you have recovered, [and because] you can’t go home there is no hope, and only death can set you free. The difference is that, in a hospital, you die quickly; here the suffering never ends. […] Family is a fundamental value to me and in my life it is all*.

Tommaso, a 31-year-old man with four children and had been imprisoned under high-security conditions when he was 22-years-old, said:

*Sometimes you forget who you are because you lose yourself, and you start asking yourself why you got a life sentence. Who am I? In this condition, you reach the point where you wonder if they sentenced the right person. You don’t recognise yourself as the same person you were twenty years ago. Now, I understand that I had an awful life. When I was younger, I wasn’t able to think, I was arrogant, but now I’m another person. Why do I have to stay here for the errors of an individual that I’m not anymore? […] What I would like to communicate to others is my true identity, my most sincere identity, which is no longer the same as before. I want to let them know that I am now a new person who knows the value of respect for others and for myself. […] Life sentence prevents you from changing your identity, keeps you always the same, fixes you on your mistake and that is not your real identity, the one you can tell others.*

Similar words were expressed by Francesco, a 42-year-old man who was arrested when he was 22 and now collaborates with a cooperative inside the prison. He has not left prison since 1999, and he speaks with his family two or three times per year. ‘I am a young man; give me a second chance. I was young and I am ashamed to return to the past. I think differently and I burned the flower of my youth. Give me this chance’.

In addition, Marcello, a 52-year-old man who, during his detention, obtained several master’s degrees and even a law degree, and who is currently working for a cooperative, stressed that becoming a criminal is not a choice, but a consequence of the social context in which a person is born: ‘There’s no human agency; there are causes and contributory causes that lead to crime. For example, if you were born in a certain suburb, you have more opportunity to become a criminal. It’s not the single human being, it’s the place where you were born. There are no criminal choices; nobody wants to become a criminal’.

### 3.2. Life Sentence as Annihilation of Life Meaning and of the Values of Generativity and Family

The second theme involved the fading of any sense of life and its value because of the life sentence. Santino, a man who spent the last 20 years in prison claiming to be innocent, affirmed the following: 

Here, life has no sense. I can survive thanks to my children and to God, who give me the strength to face each new day of my nonsensical existence. After twenty years, you lose everything: family, friends. You lose all reference to the world and people. It’s not just you who suffers, it’s your family too, because they are often forced to give away an entire part of their life in order to follow the relocations to different prisons! Not only are you no longer worth anything to society, but neither are your family members. 

Carlo continued in this vein: ‘My daughter follows me and has always followed me in this path, and she had to suffer the consequences in a physical way. She is somatising the suffering of not being able to stop anywhere to meet me. All this means that, not only am I being punished by being forced to stay in prison, but, since my children are my greatest treasure, I am causing them to suffer, and seeing them suffer because of me’. Tommaso described the same complexity that his sons experienced: ‘My family is my only wealth, and I suffer deeply because, now, even my children have to serve the penalty of having a father in prison. My daughter is now anorexic. She cannot stop crying and continuously asks her mother when Dad will come home again. She cannot accept that I am here’. 

Paul expressed a similar sentiment: ‘The family is everything for me; it is the oxygen for my life’ as did Angiolino, a man in his fifties who had been in detention for 23 years, and who divorced his wife to allow her to be free: ‘I have the bad luck, or I’m so lucky, to have a family that has always cared about me—particularly my daughters, who [also] live this sentence. My life sentence is also theirs. When I was sentenced to life in prison, my family was, too’. Angiolino described the choice to divorce his wife in this way: ‘When they issued the sentence in 2003, which became a final one in 2007 when the Court of Appeals confirmed my life sentence, my daughters’ mother and I decided that she would go on with her own life because I couldn’t give her anything more’.

Tommaso, who considered his penalty as total nonsense, said: ‘I will soon be a grandfather and father again, and this makes me happy, but this condition robs me of all chances. They don’t leave hope for anything. When you are under high security, you see other people who have been there for a long time, and you ask yourself if you will ever come out from there again. I don’t know how to live without my family’. 

Another important dimension that gives hope is faith. Santino, who jeopardised his life through a long hunger strike, linked the narrative of family to the value of religion: ‘It is hard to understand me; you should be inside me. God gives me the power, helps me to keep up my hope, even if I sometimes get angry with Him, like a son gets angry with his dad. I ask Him: you know my heart; how can you allow all this? Then I start reflecting about all the people in the world who never hurt anyone but still suffer, like children in Africa, and I console myself. Maybe it is God’s intention’. Referring to his daughters, Santino further reported: ‘Their mom and I decided that she shouldn’t bring them to the prison anymore because, when they go back home, they start to misbehave; I had hoped to obtain permission to meet them outside and with fewer limitations. Unfortunately, this did not happen, and things got more complicated’.

## 4. Discussion

From the analysis of the interview texts, two themes emerged: the values of freedom, self-consciousness and education and their failure in prison, and life sentence as annihilation of life meaning and of the values of generativity and family. The two themes showed how life imprisonment, as it is regulated and applied in the Italian legal system, placed great limitations on participants with respect to their personal values. In particular, the barriers to the intrinsic values proposed by Schwartz [20] emerged several times. The first of these, as Carlo reported, was the obstruction of personal freedom: prisoners could no longer decide anything for themselves and for others, and their privacy and self-direction were severely limited. In fact, due to the complete limitation of prisoners’ freedom in Italian prisons, they are required to obtain a permit to do any activity—be it for leisure, treatment, education or work—inside or outside the prison house. 

The value of knowledge and education was also emphasised. For example, Paul recounted his experience of imprisonment in Belgium and how re-education provides an individual with the existential motivation to become a better person. In his opinion, this form of intervention allows prisoners to develop confidence in institutions and, eventually, in all society. The Italian penitentiary health system often does not meet prisoners’ developmental needs. This goes against the declarations on health and social inequality made by the World Health Organisation and the Italian Constitution. Furthermore, severe prison conditions do not reduce the likelihood of recidivism, but rather increase post-release criminal activity [42].

In addition, the impossibility of self-direction and the inhibition of generativity were highlighted several times by participants, who found it difficult to imagine and plan their life and thus lend new meaning in it. The humiliation entailed by these obstructed values jeopardised prisoners’ mental health [43]. In fact, the negation of the possibility of changing because of life sentence inspired a sort of resignation: no past can be valorised, because no future exists, and the past self is questioned such that there is a disconnect between the self, resulting in clear difficulties in potential for perception of generativity. From this perspective, Tommaso said that his life sentence closed all the doors to his desired future, leaving no more hope for anything. He also pointed out that the knowledge of being inside a prison for life leads to anger and prevents prisoners from becoming better people. Carlo questioned his sense of self to the point of perceiving a clear disconnect between in past “self” and current sense of self such that the wrong ‘self’ was being punished. Furthermore, the annihilation of meaning in life involves one’s family and one’s relationships with loved ones, as any contact with them is regulated by magistrates. Participants perceived this condition as a particular form of abandonment, because their companions and wives were left to manage the family autonomously, without involving them in any decision. Despite this situation, family remained central to prisoners’ values [44,45,46,47,48]. 

Contact with relatives restores in prisoners a personal sense of dignity, and this has a significant positive impact on their rehabilitation and reintegration into society as well as the prevention of recidivism [44,45,46,47,48]. From a Dignity Therapy model, this connection with meaningful persons in their life has the potential to restore dignity during incarceration, or lead to greater sense of loss of meaning and dignity if not appropriately addressed by the correctional system. Indeed, maintaining family ties whilst in prison enables prisoners to improve their psychological equilibrium and reduces recurrence after the sentence [44,45,46,47,48]. The positive effect of the family value is also evidenced by the fact that children who maintain positive contact with their imprisoned fathers exhibit greater well-being than those who do not [48]. Indeed, the prisoners’ perception of the annihilation of their relationship with family disrupted any sense of generativity, that is, the value of being able to provide guidance to offspring. The continuity of the self with respect to generative aims is endangered by the life sentence. This is because the guiding role established prior to the sentence becomes severely limited after the sentence, sometimes existing only within the prisoners’ memories. In fact, the life sentence jeopardises their motivation to live; thus, it is often described as a punishment worse than the death penalty. Furthermore, this disruption of all the fundamental values that provided meaning in life was compared to death itself, to a terminal illness, to torture and to a pain that grows over the years, with the awareness that, despite the passing of time, you will not have the opportunity to return to your loved ones and to a free life.

Finally, out of this study has emerged a complex perspective on legacy among prisoners serving life sentences. The participants perceived that the transmission of their ultimate legacy’ was somehow inauthentic because they were unable to see their values being respected in practice. Because their perception of identity, integrity and dignity was closely tied to their personal values being annihilated in prison, legacy seemed to have the potential to be, on the one hand, a request that they would like to see their values being respected and, on the other hand, their denunciation of society, which makes it impossible for them to change the negative identity of the past. The recognition of the fundamental values that make up their identity leads to representing life imprisonment as slow mortification of the very meaning of life. This makes it difficult for them to pass on to others some universal meaning, if not that of the value of freedom that they are definitively denied.

## 5. Conclusions

By utilising the DT interview approach, our study was able to retrace the participating prisoners’ universe of life values. These prisoners clearly expressed how the life sentence limits the values of freedom, self-awareness and education, thus causing the annihilation of life meaning and the endangerment of psychological equilibrium. Given that all these factors are important for preserving dignity, we can evidently say that the life sentence de-humanises prisoners. The actual conditions of detention in Italian prisons are very different from those promoted by human rights watch groups and the Italian Constitution. 

According to Western culture, which is based on the value of dignity, prisons should provide concrete tools to prisoners in order to maintain their human identity and respect their personal values. Indeed, article 27 of the Italian Constitution asserts the absolute value of personal identity and dignity. As our study indicates, life imprisonment deprives prisoners of all these by destroying any dimension of existential values. All participants valorised the right to work, the right to health, the right to education and, above all, the right to love. Indeed, the penitentiary system should respect all these values, because doing so empowers prisoners to maintain family and emotional relationships as well as pursue education and the furtherance of job prospects and social reintegration, which are all values that lie at the heart of human dignity.

## 6. Limitations and Future Research

Since audio-recording was not possible inside the prison setting, it is plausible that details which would have been detectable by listening to the interviews may have been missed. Further, the interviews were performed exclusively in an Italian prison, which is considered to be one of the best institutions in terms of treatment activities and job opportunities for prisoners. To achieve greater validity in our analysis, it would have been more useful to carry out interviews with people in other prisons who are serving their sentences under harsher conditions. Future research may seek to carry out an analysis on interviews with people in persons serving life-sentence in heterogenous prison settings including those with harsher prison terms in order to increase generalizability of the data. Although qualitative methods are widely used in the field of well-being and male health [49,50], quantitative studies could be carried out to verify the self-perception of dignity in this population and to probe their universe of values.

## Figures and Tables

**Table 1:** Questions of Dignity Therapy.

1. “Tell me a little about your life history, particularly the parts that you either remember most, or think are the most important.”
2. “When did you feel most alive?”
3. “Are there specific things that you would want your family to know about you, and are there particular things you would want them to remember?”
4. “What are the most important roles you have played in life (family roles, vocational roles, community service roles, etc.)?”
5. “Why were they so important to you, and what do you think you accomplished in those roles?”
6. “What are your most important accomplishments, and what do you feel most proud of?”
7. “Are there particular things that you feel still need to be said to your loved ones, or things that you would want to take the time to say once again?”
8. “What are your hopes and dreams for your loved ones?”
9. “What have you learned about life that you would want to pass along to others? What advice or words of guidance would you wish to pass along to your (son, daughter, husband, wife, parents, others)?”
10. “Are there words or perhaps even instructions you would like to offer your family to help prepare them for the future?”
11. “In creating this permanent record, are there other things that you would like included?”
